# The Relationship Between Extended Work Hours and Turnover Among Nursing Home Staff in Nursing Homes

**DOI:** 10.1093/geroni/igaf122.1497

**Published:** 2025-12-31

**Authors:** Ganisher Davlyatov, Gregory Orewa, Rohit Pradhan

**Affiliations:** The University of Oklahoma, Oklahoma City, Oklahoma, United States; The University of Texas at San Antonio, San Antonio, Texas, United States; Texas State University, San Marcos, Texas, United States

## Abstract

High turnover rates among nursing staff including registered nurses (RNs), licensed practical nurses (LPNs), and certified nursing assistants (CNAs) have been a long-standing concern in nursing homes (NHs). Nursing staff turnover has been associated with poorer NH quality outcomes such as a higher number of quality of care deficiencies and increased mortality among residents. This study examined the association between extended work hours among nursing staff and NH turnover. It utilized multiple secondary datasets, including the Payroll-Based Journal and Care Compare: Five-Star Quality Rating System (Five-Star QRS) (2023). The dependent variable was nursing staff (RNs, LPNs, CNAs) turnover calculated by dividing the number of staff who left during the year by the average number of staff employed. The independent variable was extended work hours, measured as the percentage of nursing staff exceeding 50 work hours per week, averaged across the year to calculate the annual facility-level rate. A mixed-effect maximum likelihood regression was conducted with appropriate control variables (14,279). Results indicated that extended work hours significantly increased turnover for RNs (β = 0.075, p < 0.001) and LPNs (β = 0.091, p < 0.001), but not for CNAs. Policy and managerial efforts should focus on nursing staff retention and recruitment though workload regulations may be required to ensure sustainable, high-quality NH care. These findings highlight the need to regulate excessive work hours to reduce nursing staff turnover. Administrators should implement workload management strategies, such as capping overtime and ensuring adequate staffing, to improve retention and maintain care quality in NHs.

